# Association of commission on cancer accreditation with receipt of guideline‐concordant care and survival among patients with colon cancer

**DOI:** 10.1002/wjs.12391

**Published:** 2024-11-07

**Authors:** Kelley Chan, Bryan E. Palis, Joseph H. Cotler, Lauren M. Janczewski, Ronald J. Weigel, David J. Bentrem, Clifford Y. Ko

**Affiliations:** ^1^ American College of Surgeons Cancer Programs Chicago Illinois USA; ^2^ Department of Surgery Stritch School of Medicine Loyola University Chicago Maywood Illinois USA; ^3^ Department of Surgery Northwestern University Feinberg School of Medicine Chicago Illinois USA; ^4^ Department of Surgery Carver College of Medicine University of Iowa Iowa City Iowa USA; ^5^ Department of Surgery David Geffen School of Medicine University of California Los Angeles Los Angeles California USA

**Keywords:** colorectal, oncology, outcomes

## Abstract

**Background:**

Guideline‐concordant care (GCC) is associated with improved survival for patients with cancer; however, variations in receipt of GCC remain a concern. The objective of this study was to evaluate the association of Commission on Cancer (CoC) hospital accreditation status with receipt of GCC and survival among patients with colon cancer.

**Methods:**

This retrospective observational study identified patients diagnosed with stage I–IV colon cancer from 2018 to 2020 from the National Program of Cancer Registries and Surveillance, Epidemiology, and End Results Program Database. Guideline concordance was defined as receipt of stage‐appropriate lymphadenectomy or chemotherapy. Multivariable logistic regression models investigated associations with receipt of GCC. Cox proportional hazards regression models assessed 3‐year cancer‐specific mortality risk.

**Results:**

Of 222,583 patients with colon cancer, 146,629 (91.2%) of eligible patients received guideline‐concordant lymphadenectomy and 70,586 (81.9%) of the eligible patients received guideline‐concordant chemotherapy. Treatment at CoC‐accredited hospitals was the strongest modifiable predictor for receipt of guideline‐concordant lymphadenectomy (odds ratio [OR] 1.82; 95% confidence interval [CI] 1.75–1.88) and chemotherapy (OR 2.14; 95% CI 2.06–2.23). Among patients treated at CoC‐accredited hospitals, risk adjusted mortality was decreased for patients with stage I–II disease (hazard ratio [HR] 0.94; 95% CI 0.80–0.99), stage III disease (HR 0.93; 95% CI 0.88–0.98), and stage IV disease (HR 0.88; 95% CI 0.84–0.92).

**Conclusions:**

For patients with colon cancer, treatment at CoC‐accredited hospitals was associated with increased receipt of GCC and decreased mortality risk. Benchmarking data may serve as a valuable accountability tool for quality assessment to improve cancer treatment and outcomes.

## 1 INTRODUCTION

Despite the overall decline in mortality for patients with colon cancer in the United States, there remain significant disparities in progress observed nationally, which may reflect differences in access to and receipt of high‐quality cancer treatment.^1^ National clinical guidelines were designed to promote standardization of care, minimize variability in treatment, and to improve quality of care.^2^ Receipt of guideline‐concordant care (GCC) has been associated with improved survival for patients with colon cancer.^3^ However, significant variations in the receipt of GCC, associated with geographic and sociodemographic factors, have been described.^4^, ^5^, ^6^ It is worth noting that in a report outlining recommendations for optimizing the delivery of cancer care, the Institute of Medicine endorsed the measuring, monitoring, and comparative benchmarking of care using a core set of quality measures and recommended assessments of potential quality indicators such as hospital accreditation status.^7^

Hospital accreditation encompasses the principle that adherence to evidence‐based standards translates to high‐quality care and improved patient outcomes. The American College of Surgeons (ACS) Commission on Cancer (CoC) assesses the quality of its 1450 CoC‐accredited hospital cancer programs on an annual basis, representing over 70% of cancer patients treated in the United States annually, using quality measures concordant with national evidence‐based guidelines.^8^ CoC‐accredited hospitals are required to submit cancer registry data to the National Cancer Database (NCDB), which analyzes hospital‐level compliance of quality measures to provide benchmarking data.^8^ Currently, the NCDB is the only cancer registry that provides benchmarking data to its hospitals, which serves as a valuable accountability tool for quality assessment and improvement.^9^

Observational studies using NCDB data have shown improved compliance with quality measures and survival over time in CoC‐accredited hospitals.^8^, ^10^ However, the evaluation of performance across quality measures at CoC‐accredited hospitals compared to non‐CoC‐accredited hospitals has been limited to analyses using single state cancer registries or Medicare beneficiaries.^11^, ^12^, ^13^, ^14^ Thus, the objective of the study was to evaluate the association of treatment at CoC‐accredited hospitals, compared to non‐CoC‐accredited hospitals, with receipt of GCC and cancer‐specific survival for patients with colon cancer on a national scale.

## 2 METHODS

### 2.1 Data source

This retrospective observational study queried the United States Cancer Statistics (USCS), the official Federal cancer statistics, which includes cancer registry data from the Centers for Disease Control and Prevention (CDC) National Program of Cancer Registries (NPCR) and the National Cancer Institute (NCI) Surveillance, Epidemiology, and End Results (SEER) Program for patients diagnosed with colon cancer between January 1, 2018 and December 31, 2020.^15^ The USCS captures data on patient demographics, tumor characteristics, and treatment from all 50 states and covers 97% of the cancer population.^16^ This study was considered nonhuman subject research and exempt from Institutional Review Board review. This retrospective cohort study followed the REporting of studies Conducted using Observational Routinely collected health Data (RECORD) reporting guidelines.^17^

### 2.2 Study population

Patients aged 18 years or older with newly diagnosed colon cancer with epithelial tumor histology were included. All cases were abstracted according to the instructions and coding definitions of the North American Association of Central Cancer Registries.^18^ Cases included in the study were diagnosed and treated at reporting hospitals identified as non‐CoC‐accredited or CoC‐accredited at the time of data abstraction. Analytic cases prepared at CoC‐accredited hospitals included cases where all or part of first course treatment was performed at the reporting facility (Class of Case = 10–22).^18^ Cases were excluded from the study if abstracted from a death certificate, if first course of therapy was unknown, if none of the first course of treatment was given at the reporting hospital (Class of Case = 00), or if data on race, ethnicity or stage were missing. A majority of central cancer registries utilize probabilistic record linkage with public and private health insurance claims to extract treatment information.^19^, ^20^, ^21^

### 2.3 Variables

Patient‐level variables included age at diagnosis (years), sex, race and ethnicity, insurance status, geographic location, rural/urban/metropolitan location, and census tract poverty indicator. Tumor‐level characteristics included primary site, histology, pathological grade, stage, evidence of lymphovascular invasion, and presence of microsatellite instability. Stage was determined by the NCI SEER Summary Stage converted to the American Joint Committee on Cancer eighth edition manual.^22^, ^23^, ^24^ Outcome variables included treatment given, cancer‐specific mortality, and survival (months). The only hospital‐level variable available was CoC accreditation status at the time of case abstraction.

### 2.4 Outcomes and definitions

The primary outcomes of the study included an evaluation of receipt of GCC and mortality. Eligibility and receipt of GCC were defined according to the National Comprehensive Cancer Network (NCCN) treatment guidelines.^25^ Guideline‐concordant lymphadenectomy was defined by a minimum of 12 lymph nodes examined for patients with stage I–III disease who received surgery. Guideline‐concordant chemotherapy was defined as chemotherapy administration for patients under 80 years of age with stage III–IV disease. Patients were considered ineligible for GCC if the treatment was not part of the planned first course of therapy, they died prior to surgery and/or chemotherapy, surgery and/or chemotherapy was contraindicated due to patient factors, it was unknown why they did not receive treatment, or they refused surgery and/or chemotherapy. Mortality included an evaluation of 3‐year cancer‐specific mortality, which was based on the number of months between the date of diagnosis and the date of last contact or death. Patients were excluded if they survived less than 3 months after their cancer diagnosis.

### 2.5 Statistical analysis

Chi‐square and *t*‐test statistics were used to compare categorical and continuous characteristics, respectively, for patients treated at CoC‐accredited hospitals compared to non‐CoC‐accredited hospitals. Multivariable logistic regression models were performed to investigate factors associated with receipt of GCC, adjusting for patient sociodemographic characteristics, primary site, clinical stage, and hospital accreditation. Three‐year survival estimates were obtained from Kaplan–Meier survival curves, utilizing the logrank and Wilcoxon rank sum tests to assess survival for patients diagnosed in 2018 having at least 36 months of accrued follow‐up time. Multivariable risk adjusted cancer‐specific survival hazard ratios (HRs) were calculated with fixed‐effects Cox proportional hazards regression models. All analyses were conducted using SAS 9.4 statistical software (SAS Institute, Inc., Cary, NC, US) using a significance level of *p* < 0.05.

### 2.6 Sensitivity analysis

Recognizing the potential for differences in guideline recommendations, models investigating receipt of GCC and cancer‐specific mortality were repeated for patients with high‐risk stage II disease. High‐risk stage II disease was defined according to the NCCN treatment guidelines as patients with microsatellite stable or mismatch repair proficient stage II disease with poorly differentiated/undifferentiated histology, lymphovascular invasion, or inadequately sampled nodes (<12 lymph nodes).^25^ Additionally, a sensitivity analysis was performed for receipt of GCC for patients from nonmetropolitan locations or with low socioeconomic status (SES), defined as residing in a census tract with 20%–100% poverty, to evaluate whether treatment concordance was associated with rurality or SES.

## 3 RESULTS

There were 234,266 eligible patients, of which 11,683 were excluded due to missing data on race, ethnicity or stage, abstraction from a death certificate, or unknown first course of therapy. Of 222,583 patients included in this study, the median age was 69 years (interquartile range [IQR] 59–78 years), 119,450 (51.0%) were male, and 158,549 (71.2%) were treated at CoC‐accredited hospitals (Table 1). Patients treated at CoC‐accredited hospitals were more likely to be diagnosed with higher grade and stage disease and receive treatment for their cancer. Among those who were treated at non‐CoC‐accredited hospitals, a higher proportion of patients were less likely to receive treatment for their cancer and were more likely to have their death attributable to their cancer. Surgery and chemotherapy treatment summaries are reported in Table S1. Median regional lymph nodes examined were 18 (IQR 14–24) and 19 (IQR 15–26) for non‐CoC‐accredited hospitals and CoC‐accredited hospitals, respectively. A higher proportion of patients at CoC‐accredited hospitals had ≥22 lymph nodes examined (Table 1). Lymph node examination by primary site are noted in Table S2. Among patients with stage I–III disease, 97.6% of those treated at CoC‐accredited hospitals and 96.1% of those treated at non‐CoC‐accredited hospitals received surgery. Of the patients eligible for GCC, 146,629 (91.2%) patients received guideline‐concordant lymphadenectomy and 70,586 (81.9%) received guideline‐concordant chemotherapy. Receipt of GCC was higher for both quality measures among patients treated at CoC‐accredited hospitals compared to non‐CoC‐accredited hospitals (Table 2).

**TABLE 1:** Sociodemographic and tumor characteristics of patients diagnosed with colon cancer by hospital accreditation status, 2018–2020.

	Non‐CoC‐accredited, *n* (%)	CoC‐accredited, *n* (%)	*p*‐value
Total	64,034 (28.8)	158,549 (71.2)	
Age (years)			<0.001
Median (IQR)	69 (60–78)	69 (59–78)	
18–49	5286 (8.3)	15,552 (9.8)	
50–59	10,461 (16.3)	27,490 (17.3)	
60–69	16,667 (26.0)	40,427 (25.5)	
70–79	17,598 (27.5)	41,614 (26.3)	
≥80	14,022 (21.9)	33,466 (21.1)	
Sex			0.012
Male	32,897 (51.4)	80,506 (50.8)	
Female	31,137 (48.6)	78,043 (49.2)	
Race and ethnicity			<0.001
NH White	47,059 (73.5)	116,158 (73.3)	
NH Black	7790 (12.1)	21,303 (13.4)	
NH American Indian/Alaska Native	622 (1.0)	820 (0.5)	
NH Asian or Pacific Islander	2014 (3.2)	6208 (3.9)	
Hispanic (all races)	6549 (10.2)	14,060 (8.9)	
Geographic location			<0.001
New England	1670 (2.6)	5954 (3.8)	
East North Central	8662 (13.5)	26,528 (16.7)	
East South Central	4826 (7.5)	11,717 (7.4)	
Middle Atlantic	7309 (11.4)	23,969 (15.1)	
Mountain	5845 (9.1)	6838 (4.3)	
Pacific	9055 (14.1)	21,908 (13.8)	
South Atlantic	12,885 (20.1)	35,320 (22.3)	
West North Central	3547 (5.5)	10,202 (6.4)	
West South Central	10,235 (16.0)	16,113 (10.2)	
Insurance			<0.001
Not insured	1520 (2.4)	4083 (2.6)	
Private	14,939 (23.3)	40,997 (25.9)	
Medicaid	3397 (5.3)	9476 (6.0)	
Medicare	30,975 (48.4)	78,107 (49.3)	
Other government	1223 (1.9)	2245 (1.4)	
Unknown	11,980 (18.7)	23,641 (14.9)	
Rural/urban			<0.001
Metropolitan	48,506 (75.8)	134,462 (84.8)	
Urban	13,928 (21.8)	21,103 (13.3)	
Rural	1600 (2.5)	2984 (1.9)	
Census tract poverty indicator			<0.001
0%–5% poverty	8593 (13.4)	28,941 (18.3)	
5%–<10% poverty	15,514 (24.2)	40,984 (25.9)	
10%–<20% poverty	20,674 (32.3)	45,915 (29.0)	
20%–100% poverty	13,294 (20.8)	29,346 (18.5)	
Unknown	5959 (9.3)	13,363 (8.4)	
Stage			<0.001
I	23,356 (36.5)	52,012 (32.8)	
II	10,958 (17.1)	26,926 (17.0)	
III	16,878 (26.4)	43,797 (27.6)	
IV	12,842 (20.1)	35,814 (22.6)	
Primary site			<0.001
Right colon	30,593 (47.8)	76,609 (48.3)	
Transverse colon	6718 (10.5)	16,964 (10.7)	
Left colon	23,012 (35.9)	57,564 (36.3)	
Overlapping lesion of colon and colon NOS	3711 (5.8)	7412 (4.7)	
Histology			0.123
Adenocarcinoma	58,640 (91.6)	144,945 (91.4)	
Mucinous adenocarcinoma	3835 (6.0)	9836 (6.2)	
Other	1559 (2.4)	3768 (2.4)	
Grade pathological			<0.001
Well differentiated	6940 (10.8)	14,099 (8.9)	
Moderately differentiated	35,376 (55.3)	91,704 (57.8)	
Poorly differentiated	8730 (13.6)	23,308 (14.7)	
Undifferentiated	223 (0.4)	621 (0.4)	
GX, unknown	12,765 (19.9)	28,817 (18.2)	
Lymphovascular invasion			<0.001
Not present/Not identified	33,864 (52.9)	84,841 (53.5)	
Present/Identified	14,987 (23.4)	43,306 (27.3)	
Unknown	15,183 (23.7)	30,402 (19.2)	
Microsatellite instability			<0.001
MSI stable	24,047 (37.6)	81,351 (51.3)	
MSI unstable low	1471 (2.3)	4243 (2.7)	
MSI unstable high	5837 (9.1)	18,876 (11.9)	
Unknown/not documented	32,679 (51.0)	54,079 (34.1)	
Treatment given			<0.001
No treatment given	4420 (6.9)	7391 (4.7)	
Treatment given	59,528 (93.0)	150,963 (95.2)	
Active surveillance	86 (0.1)	195 (0.1)	
Regional lymph nodes examined for stage I–III (nodes)	*n* = 46,015	*n* = 114,700	<0.001
Median (IQR)	18 (14–24)	19 (15–26)	
<12	5885 (12.8)	8201 (7.1)	
12–21	24,290 (54.2)	60,334 (52.6)	
≥22	15,210 (33.0)	46,165 (40.3)	
Cause of death if applicable			<0.001
Attributable to this cancer	10,639 (59.0)	25,871 (57.9)	
Other cause of death	6913 (38.3)	17,905 (40.0)	
Cause missing/unknown	487 (2.7)	941 (2.1)	

Abbreviations: CoC, Commission on Cancer; GX, grade cannot be assessed; IQR, interquartile range; MSI, microsatellite instability; NH, non‐Hispanic; NOS, not otherwise specified; STD, standard deviation.

**TABLE 2:** Percentage of cases concordant with the lymphadenectomy and chemotherapy quality measures by hospital accreditation status.

	Cases concordant with quality measure, *n* (%)			*p*‐value
	Lymphadenectomy for stage I–III	Chemotherapy for stage III–IV	Lymphadenectomy and chemotherapy for stage III	
Non‐CoC‐accredited	40,130 (87.2)	16,718 (72.0)	8628 (67.7)	<0.001
CoC‐accredited	106,499 (92.9)	53,868 (85.5)	27,939 (83.2)	<0.001

Abbreviation: CoC, Commission on Cancer.

Treatment at CoC‐accredited hospitals, compared to non‐CoC‐accredited hospitals, was associated with increased receipt of GCC for both quality measures (Table 3). For patients treated at CoC‐accredited hospitals, the adjusted odds ratio (OR) for receipt of guideline‐concordant lymphadenectomy during surgery was 1.82 (95% confidence interval [CI] 1.75–1.88) and for guideline‐concordant chemotherapy was 2.14 (95% CI 2.06–2.23). Non‐Hispanic Black patients were less likely to receive guideline‐concordant lymphadenectomy and chemotherapy.

**TABLE 3:** Multivariable adjusted odds ratio for receipt of guideline concordant lymphadenectomy and chemotherapy measures.

	Lymphadenectomy for stage I–III, OR (95% CI)	*p*‐value	Chemotherapy for stage III–IV, OR (95% CI)	*p*‐value
Sex				
Male	Ref.		Ref.	
Female	1.15 (1.11–1.20)	<0.001	1.06 (1.03–1.11)	0.001
Age				
18–49	1.92 (1.76–2.09)	<0.001	2.08 (1.93–2.23)	<0.001
50–59	1.17 (1.10–1.24)	<0.001	1.39 (1.32–1.47)	<0.001
60–69	Ref.		Ref.	
70–79	0.96 (0.92–1.01)	<0.001	0.62 (0.60–0.65)	<0.001
≥80	0.82 (0.77–0.86)	<0.001	N/A	
Insurance status				
Medicare	Ref.		Ref.	
Private	1.12 (1.06–1.18)	0.003	1.39 (1.32–1.47)	<0.001
Medicaid	1.03 (0.94–1.12)	0.702	0.97 (0.89–1.05)	0.716
Not insured	1.04 (0.92–1.18)	0.997	0.76 (0.69–0.84)	<0.001
Other government	1.03 (0.89–1.19)	0.846	0.96 (0.83–1.10)	0.999
Unknown	1.03 (0.98–1.09)	0.782	0.78 (0.74–0.82)	<0.001
Race and ethnicity				
NH White	Ref.		Ref.	
NH Black	0.80 (0.75–0.84)	<0.001	0.83 (0.79–0.88)	0.002
NH American Indian/Alaska Native	0.85 (0.70–1.04)	0.670	0.91 (0.74–1.12)	0.982
NH Asian or Pacific Islander	0.94 (0.85–1.04)	0.159	0.95 (0.86–1.04)	0.471
Hispanic (all races)	0.84 (0.79–0.90)	<0.001	0.89 (0.84–0.95)	<0.001
Rural/urban				
Metropolitan	1.41 (1.34–1.47)	<0.001	0.88 (0.84–0.93)	<0.001
Urban	Ref.		Ref.	
Rural	1.01 (0.90–1.13)	0.004	1.07 (0.93–1.23)	0.062
Census tract poverty indicator				
0%–5% poverty	1.47 (1.38–1.57)	<0.001	1.64 (1.53–1.75)	<0.001
5%–<10% poverty	1.28 (1.21–1.35)	<0.001	1.44 (1.36–1.52)	<0.001
10%–<20% poverty	1.16 (1.10–1.22)	<0.001	1.23 (1.16–1.29)	<0.001
20%–100% poverty	Ref.		Ref.	
Unknown	1.31 (1.22–1.41)	0.018	1.46 (1.35–1.57)	0.002
Hospital accreditation				
Non‐CoC‐accredited	Ref.		Ref.	
CoC‐accredited	1.82 (1.75–1.88)	<0.001	2.14 (2.06–2.23)	<0.001
Stage				
I	Ref.		N/A	
II	1.87 (1.78–1.96)	<0.001	N/A	
III	1.89 (1.82–1.97)	<0.001	Ref.	
IV	N/A		0.82 (0.79–0.85)	<0.001
Primary site				
Right colon	Ref.		Ref.	
Transverse colon	0.46 (0.43–0.48)	<0.001	0.91 (0.85–0.97)	<0.001
Left colon	0.41 (0.39–0.43)	<0.001	1.09 (1.05–1.14)	<0.001
Overlapping lesion of colon and colon NOS	0.55 (0.50–0.61)	<0.001	0.45 (0.42–0.48)	<0.001

Abbreviations: CoC, Commission on Cancer; NH, non‐Hispanic; NOS, not otherwise specified; OR, odds ratio.

**FIGURE 1:**
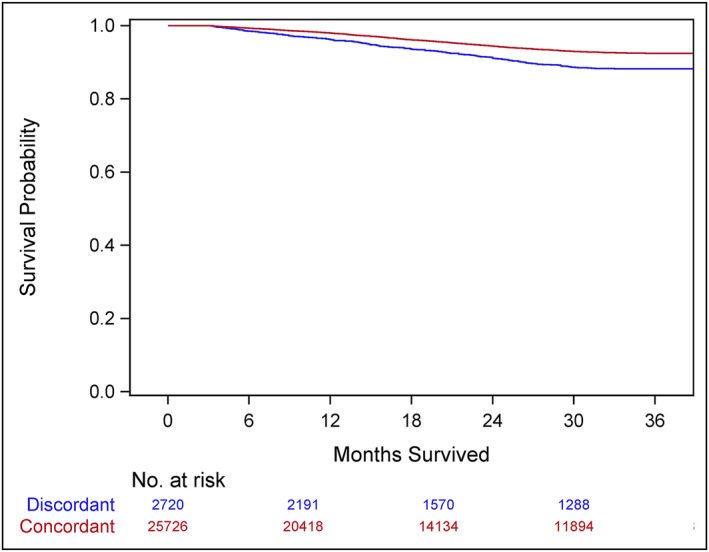
3‐year cancer‐specific survival among patients with stage I–II colon cancer diagnosed in 2018 by receipt of guideline concordant lymphadenectomy (Logrank *p* < 0.001, Wilcoxon < 0.001).

**FIGURE 2:**
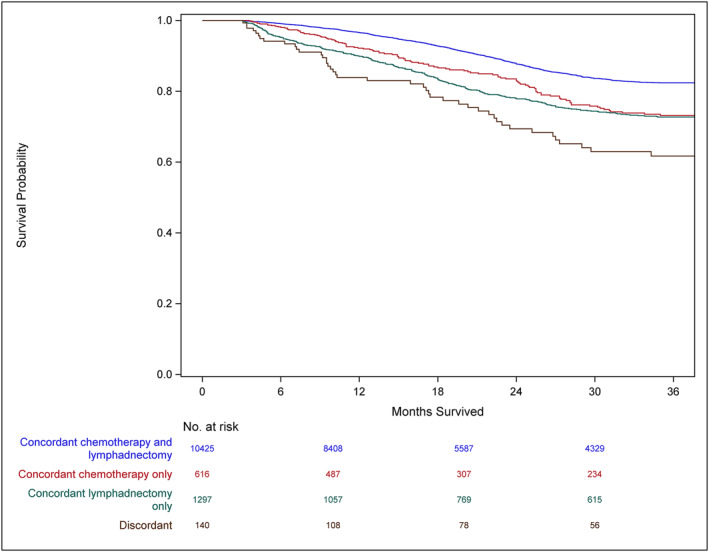
3‐year cancer‐specific survival among patients with stage III colon cancer diagnosed in 2018 by receipt of guideline concordant lymphadenectomy and chemotherapy (Logrank *p* < 0.001, Wilcoxon < 0.001).

**FIGURE 3:**
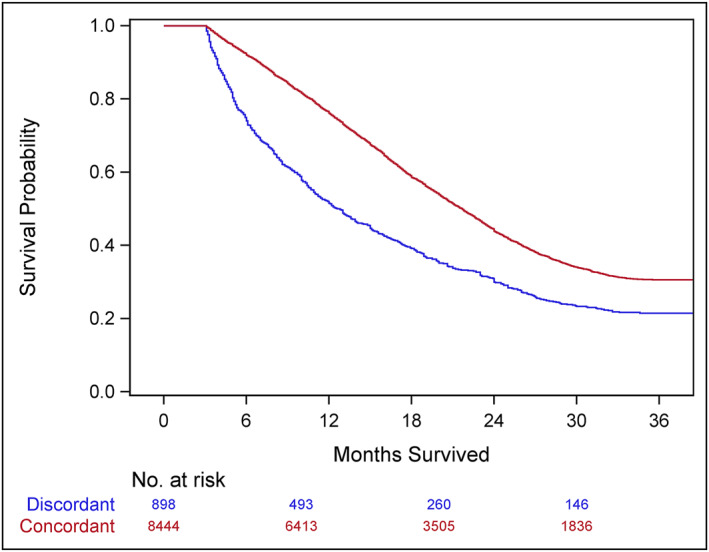
3‐year cancer‐specific survival among patients with stage IV colon cancer diagnosed in 2018 by receipt of guideline concordant chemotherapy (Logrank *p* < 0.001, Wilcoxon < 0.001).

Three‐year cancer‐specific survival was higher for patients who received GCC, compared to patients who received discordant care, for all disease stages. For patients with stage I–II disease, survival for patients who received GCC was 92.5% and for patients who received discordant care was 88.2% (Figure 1). Among patients with stage III disease, survival was 82.4% for patients who received GCC and 61.7% for patients who received discordant care (Figure 2). For patients with stage IV disease, survival for those who received GCC was 30.5% and for those who received discordant care was 21.5% (Figure 3). On multivariable analysis, treatment at CoC‐accredited hospitals was associated with decreased mortality risk for all cohorts eligible for treatment (Table 4).

**TABLE 4:** Adjusted cancer‐specific survival hazard ratios for cases diagnosed in 2018 eligible for the lymphadenectomy and chemotherapy measures.

	Stage I–II, HR (95% CI)	*p*‐value	Stage III, HR (95% CI)	*p*‐value	Stage IV, HR (95% CI)	*p*‐value
Sex						
Male	Ref.		Ref.		Ref.	
Female	0.80 (0.75–0.84)	<0.001	0.90 (0.85–0.95)	<0.001	0.94 (0.90–0.97)	0.001
Age						
18–49	0.48 (0.39–0.59)	<0.001	0.56 (0.49–0.65)	<0.001	0.76 (0.71–0.81)	<0.001
50–59	0.65 (0.57–0.74)	<0.001	0.83 (0.75–0.92)	<0.001	0.85 (0.80–0.90)	<0.001
60–69	Ref.		Ref.		Ref.	
70–79	1.65 (1.52–1.79)	<0.001	2.66 (2.46–2.88)	<0.001	1.22 (1.16–1.29)	<0.001
≥80	3.36 (3.10–3.63)	<0.001	N/A		N/A	
Insurance status						
Medicare	Ref.		Ref.		Ref.	
Private	0.68 (0.62–0.75)	<0.001	0.70 (0.64–0.76)	<0.001	0.84 (0.79–0.89)	<0.001
Medicaid	1.27 (1.10–1.47)	0.001	1.12 (0.98–1.28)	0.090	1.03 (0.95–1.12)	0.474
Not insured	0.97 (0.76–1.23)	0.065	1.18 (0.99–1.42)	0.070	0.97 (0.88–1.08)	0.612
Other government	0.86 (0.69–1.08)	0.189	0.83 (0.66–1.04)	0.109	0.81 (0.69–0.95)	0.010
Unknown	0.67 (0.62–0.72)	<0.001	0.72 (0.67–0.78)	<0.001	0.94 (0.88–1.01)	0.072
Race and ethnicity						
NH White	Ref.				Ref.	
NH Black	1.08 (0.99–1.18)	0.083	1.00 (0.92–1.09)	0.980	1.07 (1.01–1.13)	0.023
NH American Indian/Alaska Native	1.31 (0.93–1.86)	0.128	1.12 (0.82–1.53)	0.473	1.19 (0.94–1.49)	0.144
NH Asian or Pacific Islander	0.58 (0.48–0.70)	<0.001	0.66 (0.56–0.78)	<0.001	0.74 (0.65–0.84)	<0.001
Hispanic (all races)	0.76 (0.69–0.85)	<0.001	0.82 (0.74–0.91)	<0.001	0.80 (0.75–0.86)	<0.001
Rural/urban						
Metropolitan	0.96 (0.90–1.04)	0.311	0.93 (0.86–1.00)	0.052	0.95 (0.90–1.01)	0.093
Urban	Ref.		Ref.		Ref.	
Rural	0.96 (0.81–1.16)	0.694	1.02 (0.85–1.23)	0.807	0.92 (0.79–1.07)	0.268
Census tract poverty indicator						
0%–5% poverty	0.66 (0.60–0.72)	<0.001	0.72 (0.65–0.79)	<0.001	0.78 (0.73–0.84)	<0.001
5%–<10% poverty	0.75 (0.69–0.81)	<0.001	0.83 (0.77–0.90)	<0.001	0.87 (0.82–0.93)	<0.001
10%–<20% poverty	0.90 (0.83–0.96)	0.003	0.90 (0.84–0.97)	0.008	0.91 (0.86–0.97)	0.002
20%–100% poverty	Ref.		Ref.		Ref.	
Unknown	0.91 (0.81–1.03)	0.137	1.01 (0.89–1.13)	0.941	0.93 (0.85–1.01)	0.094
Hospital accreditation						
Non‐CoC‐accredited	Ref.		Ref.		Ref.	
CoC‐Accredited	0.94 (0.80–0.99)	0.037	0.93 (0.88–0.98)	0.023	0.88 (0.84–0.92)	<0.001
Stage						
I	Ref.		N/A		N/A	
II	1.48 (1.40–1.56)	<0.001	N/A		N/A	
III	N/A		N/A		N/A	
IV	N/A		N/A		N/A	
Primary site						
Right colon	Ref.		Ref.		Ref.	
Transverse colon	1.11 (1.02–1.20)	0.011	0.94 (0.86–1.02)	0.142	0.98 (0.91–1.06)	0.569
Left colon	1.11 (1.04–1.18)	0.001	0.83 (0.78–0.88)	<0.001	0.80 (0.76–0.84)	<0.001
Overlapping lesion of colon and colon NOS	1.51 (1.31–1.74)	<0.001	1.30 (1.13–1.49)	<0.001	1.61 (1.51–1.72)	<0.001

Abbreviations: CoC, Commission on Cancer; HR, hazard ratio; NH, non‐Hispanic; NOS, not otherwise specified.

Recognizing the potential for differences in guideline recommendations, models investigating receipt of GCC and cancer‐specific mortality were repeated for patients with high‐risk stage II disease. Treatment at CoC‐accredited hospitals remained an independent predictor of receipt of guideline‐concordant chemotherapy (OR 1.38; 95% CI 1.25–1.52) and decreased cancer‐specific mortality risk (HR 0.74; 95% CI 0.59–0.93) (Tables S3 and S4). Additionally, sensitivity analyses evaluating receipt of GCC among patients residing in nonmetropolitan locations or with low SES likewise showed treatment at CoC‐accredited hospitals remained associated with higher odds of receiving guideline‐concordant lymphadenectomy and chemotherapy (Table S5).

## 4 DISCUSSION

In this study, the association of CoC hospital accreditation status on GCC and contemporary colon cancer patient outcomes was evaluated on a national scale. Treatment at CoC‐accredited hospitals, compared to non‐CoC‐accredited hospitals, was found to be the strongest modifiable predictor of receipt of guideline‐concordant lymphadenectomy and chemotherapy. Cancer‐specific mortality risk was also decreased for patients treated at CoC‐accredited hospitals compared to non‐CoC‐accredited hospitals.

Patients with colon cancer treated at CoC‐accredited hospitals were more likely to be diagnosed with higher grade and stage disease compared to those treated at non‐CoC‐accredited hospitals. Prior studies comparing case counts between the NCDB's hospital‐based registry and population‐based registries have only noted variations in geographic and sociodemographic factors.^26^, ^27^ Although a recent study using the Pennsylvania Cancer Registry reported increased stage of disease for patients treated at CoC‐accredited hospitals compared to non‐CoC‐accredited hospitals, the current study describes higher disease complexity of patients with colon cancer treated at CoC‐accredited hospitals on a national scale.^28^ Patients treated at CoC‐accredited hospitals were also more likely to receive treatment compared to those treated at non‐CoC‐accredited hospitals. The Iowa Cancer Registry found that the nontreatment rate in their cancer registry was 48% higher than the rate reported in the NCDB, suggesting that nontreatment was more common in patients treated at non‐CoC‐accredited hospitals.^29^

Treatment at CoC‐accredited hospitals was the strongest modifiable predictor of receipt of GCC for patients with colon cancer. Additionally, for patients residing in nonmetropolitan locations or with low SES, treatment at CoC‐accredited hospitals remained associated with higher odds of receiving GCC. This finding is consistent with a prior report using the Iowa Cancer Registry, which demonstrated CoC‐accreditation to be the strongest predictor of hospital performance on quality measures regardless of hospital rurality and size.^11^ The authors emphasize that data reporting and feedback of guideline compliance, which are key components of CoC accreditation, have been associated with improved performance on quality measures. CoC accreditation is founded on the principle that adherence to evidence‐based standards translates to high‐quality cancer care that improves patient outcomes.

Patients who received GCC had improved survival compared to patients who received discordant care. This is consistent with prior studies demonstrating improved survival for patients with cancer receiving GCC. For example, Shulman et al. found that after introduction of the colon lymphadenectomy quality measure, performance at CoC‐accredited hospitals improved over time with paralleled improvements in survival based on hospital type.^8^ Similarly, a study using the Texas Cancer Registry found that patients with colon cancer who received stage‐specific GCC had significantly better 5‐year survival compared to those who received discordant care.^30^ The association of treatment at CoC‐accredited hospitals with decreased cancer‐specific mortality risk may represent an effect of increased delivery of GCC or may be the result of related factors such as the coordination of care provided by CoC‐accredited hospitals and increased availability of oncology‐related services compared to non‐CoC‐accredited hospitals.^31^

Cancer care is complex and multidimensional and thus the association of performance on individual quality measures with improved outcomes may be influenced by multiple factors. For example, lymph node harvest may be representative of a higher quality of surgical resection. A previous analysis of patients in the NCDB demonstrated differences in lymph node harvest for right and left colon cancer and worse survival for patients with stage II–III right sided tumors, which was improved with higher lymph node yield.^32^ In the current study, patients with right colon cancer had a higher lymph node harvest and while the median number of lymph nodes examined was not largely different, a higher proportion of patients treated at CoC‐accredited hospitals had a lymph node harvest ≥22. This increased lymph node harvest may be secondary to a more thorough cancer operation including lymphadenectomy en bloc with proximal vascular ligation.^33^ It is worth noting that the ACS has published operative standards to define critical elements of optimal cancer surgery to improve surgical quality and outcomes and has implemented synoptic reporting standards tied to CoC accreditation.^34^, ^35^ Additionally, increased lymph node harvest may increase the odds of stage migration, which may influence the differences observed in outcomes for stage II and stage III patients.^36^ Further studies are warranted to investigate postoperative prognosis and decisions for adjuvant chemotherapy independent of stage migration.

Limitations in the current study must be acknowledged in making comparisons between CoC‐accredited and non‐CoC‐accredited hospitals. First, data rely on cancer registry staff and are subject to reporting and abstracting error. Second, data on patient comorbidities, postoperative complications, stage migration, hospital program type or volume, and delays in treatment were not available. Additionally, data on the intention of treatment, including palliative or curative intent, was not available. It is possible that these factors may have contributed to treatment decisions and may be reflected in survival differences. Third, conversion of NCI SEER Summary Stage to the American Joint Committee on Cancer eighth edition manual may be associated with misclassification of disease stage. Fourth, although cases with unknown accreditation status and cases who received no treatment at the reporting hospital were excluded, the data limit recognition of patients who received part of their treatment at a different hospital from the reporting hospital. Last, data on tumor budding, bowel obstruction, or lesions with localized perforation were not available, and the current study may underreport patients with high‐risk stage II disease. However, adjuvant chemotherapy for patients with colon cancer with obstruction or perforation has not been associated with improved recurrence‐free survival or disease‐specific survival.^37^

## 5 CONCLUSION

Despite the higher disease complexity of patients with colon cancer treated at CoC‐accredited hospitals, treatment at CoC‐accredited hospitals was associated with increased receipt of GCC and decreased cancer‐specific mortality risk. Benchmarking data may serve as a valuable accountability tool for quality assessment and improvement to improve cancer treatment and outcomes.

## AUTHOR CONTRIBUTIONS

**Kelley Chan**: Conceptualization; data curation; formal analysis; methodology; resources; validation; writing—original draft; writing—review & editing. **Bryan E. Palis**: Formal analysis; methodology; resources; writing—review & editing. **Joseph H. Cotler**: Formal analysis; methodology; resources; writing—review & editing. **Lauren M. Janczewski**: Formal analysis; methodology; writing—review & editing. **Ronald J. Weigel**: Conceptualization; investigation; methodology; supervision; writing—review & editing. **David J. Bentrem**: Investigation; methodology; supervision; writing—review & editing. **Clifford Y. Ko**: Conceptualization; investigation; methodology; supervision; writing—review & editing.

## CONFLICT OF INTEREST STATEMENT

None of the authors have conflicts of interest.

## ETHICS STATEMENT

Data within the USCS are completely de‐identified and this study was exempt from review by the American College of Surgeon's institutional review board.

## Supporting information

Supporting Information S1

## Data Availability

The data that support the findings of this study are available after approval from https://seer.cancer.gov/data/access.html with signed data access agreement. Codes are available from the corresponding author upon reasonable request.
