# Supplementary material for: Association of commission on cancer accreditation with receipt of guideline‐concordant care and survival among patients with colon cancer
Source: World J Surg. 2024 Nov 7;49(1):34–45. doi: 10.1002/wjs.12391 (PMC11711115; doi:10.1002/wjs.12391)
Supplement: Supplementary file 1 — Supporting Information S1 [file WJS-49-34-s001.docx]

**Supplementary Material**

**Association of Commission on Cancer Accreditation with Receipt of Guideline-Concordant Care and Survival Among Patients with Colon Cancer**

Kelley Chan MD;^1,2^ Bryan E Palis MA;^1^ Joseph H Cotler PhD;^1^ Lauren M Janczewski MD, MS;^1,3^ Ronald J Weigel MD, PhD, MBA;^1,4^ David J Bentrem MD, MS;^3^ Clifford Y Ko MD, MS, MSHS^1,5^

**Affiliations**

**^1^** American College of Surgeons Cancer Programs, Chicago, IL, USA

**^2^** Department of Surgery, Loyola University Chicago Stritch School of Medicine, Maywood, IL, USA

^3^Department of Surgery, Northwestern University Feinberg School of Medicine, Chicago, IL, USA

^4^Department of Surgery, University of Iowa Carver College of Medicine, Iowa City, IA, USA

^5^Department of Surgery, University of California Los Angeles David Geffen School of Medicine, Los Angeles, CA, USA

**Correspondence**:

Kelley Chan, MD

American College of Surgeons Cancer Programs, Chicago, IL

633 N. St. Clair Street, 22nd Floor, Chicago, IL 60611

kchan@facs.org

**Supplemental Table 1.** Summary of surgery and chemotherapy by stage of disease.

| **Treatment** | **Non-CoC-Accredited,**  **n (%)** | **CoC-Accredited,**  **n (%)** | **p-value** |
| --- | --- | --- | --- |
| Surgery for Stage I-III (N=166249) |  |  |  |
| Surgery performed | 46349 (96.1) | 115117 (97.6) | <0.001 |
| Not part of planned first course treatment | 1443 (3.0) | 1782 (1.5) |  |
| Contraindicated due to patient risk factors | 135 (0.3) | 432 (0.4) |  |
| Died prior to surgery | 25 (0.1) | 82 (0.1) |  |
| Refused by patient | 146 (0.3) | 455 (0.4) |  |
| Unknown reason for no treatment | 139 (0.3) | 144 (0.1) |  |
| Chemotherapy for Stage III (N=37319) |  |  | <0.001 |
| Chemotherapy, NOS | 462 (4.5) | 668 (2.5) |  |
| Chemotherapy, single agent | 796 (7.7) | 2628 (9.8) |  |
| Chemotherapy, multiple agents | 5359 (51.8) | 17503 (64.9) |  |
| Not part of planned first course treatment | 2290 (22.1) | 2953 (11.0) |  |
| Contraindicated due to patient risk factors | 120 (1.2) | 500 (1.9) |  |
| Died prior to therapy | 50 (0.5) | 196 (0.7) |  |
| Refused by patient | 356 (3.4) | 1286 (4.8) |  |
| Unknown reason for no treatment | 921 (9.0) | 1231 (4.6) |  |
| Chemotherapy for Stage IV (N=32670) |  |  | <0.001 |
| Chemotherapy, NOS | 335 (4.0) | 334 (1.4) |  |
| Chemotherapy, single agent | 365 (4.4) | 1201 (4.9) |  |
| Chemotherapy, multiple agents | 4398 (52.8) | 16364 (67.3) |  |
| Not part of planned first course treatment | 2269 (27.2) | 3632 (14.9) |  |
| Died prior to therapy | 151 (1.8) | 502 (2.1) |  |
| Contraindicated due to patient risk factors | 181 2.2) | 686 (2.8) |  |
| Refused by patient | 271 (3.3) | 993 (4.1) |  |
| Unknown reason for no treatment | 367 (4.4) | 621 (2.6) |  |

NOS = not otherwise specified, CoC = Commission on Cancer

**Supplemental Table 2.** Comparison of lymph node harvest by primary site and Commission on Cancer accreditation status for patients with stage I-III colon cancer who received surgery.

|  | **Primary Site** | **Lymph Node Harvest (nodes)** | | | **p-value** |
| --- | --- | --- | --- | --- | --- |
|  |  | **<12** | **12-21** | **≥22** |  |
| Non-CoC-Accredited  (N=46015) |  |  |  |  | <0.001 |
|  | Right colon (n=23747) | 2157 (9.1) | 12915 (54.4) | 8675 (36.5) |  |
|  | Transverse colon (n=5159) | 852 (16.5) | 2637 (51.1) | 1670 (32.4) |  |
|  | Left colon (n=15667) | 2678 (17.1) | 8631 (55.1) | 4358 (27.8) |  |
|  | Overlapping lesion of colon and colon NOS (n=1442) | 198 (13.7) | 737 (51.1) | 507 (35.2) |  |
| CoC-Accredited  (N=114700) |  |  |  |  |  |
|  | Right colon (n=58959) | 2736 (4.6) | 30414 (51.6) | 25809 (43.8) | <0.001 |
|  | Transverse colon (n=13148) | 1278 (9.7) | 6562 (49.9) | 5308 (40.4) |  |
|  | Left colon (n=39622) | 3944 (10.0) | 21878 (55.2) | 13789 (34.8) |  |
|  | Overlapping lesion of colon and colon NOS (n=2971) | 232 (7.8) | 1480 (49.8) | 1259 (42.4) |  |

NOS = not otherwise specified, CoC = Commission on Cancer

**Supplemental Table 3.** Multivariable adjusted odds ratio for receipt of chemotherapy for high-risk stage II colon cancer.

|  | **Chemotherapy Measure for High-Risk Stage II, OR (95% CI)** | **p-value** |
| --- | --- | --- |
| Sex |  |  |
| Male | Ref. |  |
| Female | 1.03 (0.95 – 1.12) | 0.465 |
| Age |  |  |
| 18-49 | 2.01 (1.73 – 2.33) | <0.001 |
| 50-59 | 1.06 (0.94 – 1.19) | 0.001 |
| 60-69 | Ref. |  |
| 70-79 | 0.81 (0.72 – 0.91) | 0.008 |
| Insurance Status |  |  |
| Medicare | Ref. |  |
| Private | 1.14 (1.02 – 1.29) | 0.029 |
| Medicaid | 1.32 (1.10 – 1.59) | 0.027 |
| Not insured | 1.56 (1.20 – 2.04) | 0.002 |
| Other government | 0.82 (0.57 – 1.18) | 0.050 |
| Unknown | 0.97 (0.85 – 1.09) | 0.059 |
| Race and Ethnicity |  |  |
| NH White | Ref. |  |
| NH Black | 1.01 (0.89 – 1.15) | 0.211 |
| NH American Indian/Alaska Native | 1.39 (0.82 – 2.37) | 0.295 |
| NH Asian or Pacific Islander | 1.28 (1.06 – 1.55) | 0.012 |
| Hispanic (All Races) | 0.94 (0.81 – 1.08) | 0.080 |
| Rural/Urban |  |  |
| Metropolitan | 0.98 (0.87 – 1.10) | 0.942 |
| Urban | Ref. |  |
| Rural | 0.97 (0.70 – 1.34) | 0.901 |
| Census Tract Poverty Indicator |  |  |
| 0-5% Poverty | 0.92 (0.80 – 1.05) | 0.966 |
| 5-<10% Poverty | 0.92 (0.81 – 1.04) | 0.896 |
| 10- <20% Poverty | 1.01 (0.90 – 1.14) | 0.860 |
| 20-100% Poverty | Ref. |  |
| Unknown | 0.75 (0.63 – 0.90) | 0.002 |
| Hospital Accreditation |  |  |
| Non-CoC-Accredited | Ref. |  |
| CoC-accredited | 1.38 (1.25 – 1.52) | <0.001 |
| Primary Site |  |  |
| Right colon | Ref. |  |
| Transverse colon | 0.95 (0.83 – 1.09) | 0.530 |
| Left colon | 0.76 (0.69 – 0.83) | <0.001 |
| Overlapping lesion of colon and colon NOS | 0.99 (0.76 – 1.28) | 0.456 |

NH = Non-Hispanic, NOS = Not otherwise specified, CoC = Commission on Cancer

**Supplemental Table 4.** Adjusted cancer-specific survival hazard ratios for patients with high-risk stage II colon cancer.

|  | **High-Risk Stage II, HR (95% CI)** | **p-value** |
| --- | --- | --- |
| Sex |  |  |
| Male | Ref. |  |
| Female | 0.77 (0.62 – 0.94) | 0.012 |
| Age |  |  |
| 18-49 | 0.59 (0.38 – 0.91) | 0.016 |
| 50-59 | 0.58 (0.43 – 0.77) | <0.001 |
| 60-69 | Ref. |  |
| 70-79 | 1.44 (1.18 – 1.76) | <0.001 |
| Insurance Status |  |  |
| Medicare | Ref. |  |
| Private | 0.65 (0.44 – 0.97) | 0.033 |
| Medicaid | 1.71 (1.04 – 2.81) | 0.033 |
| Not insured | 0.89 (0.35 – 2.23) | 0.800 |
| Other government | 95 (0.72 – 1.26) | 0.220 |
| Unknown | 0.95 (0.72 – 1.26) | 0.721 |
| Race and Ethnicity |  |  |
| NH White | Ref. |  |
| NH Black | 1.03 (0.74 – 1.43) | 0.862 |
| NH American Indian/Alaska Native | 2.02 (0.74 – 5.48) | 0.168 |
| NH Asian or Pacific Islander | 0.61 (0.32 – 1.16) | 0.130 |
| Hispanic (All Races) | 0.69 (0.46 – 1.04) | 0.076 |
| Rural/Urban |  |  |
| Metropolitan | 0.88 (0.65 – 1.18) | 0.381 |
| Urban | Ref. |  |
| Rural | 0.70 (0.32 – 1.55) | 0.382 |
| Census Tract Poverty Indicator |  |  |
| 0-5% Poverty | 0.74 (0.52 – 1.05) | 0.090 |
| 5-<10% Poverty | 0.80 (0.59 – 1.10) | 0.175 |
| 10- <20% Poverty | 0.90 (0.67 – 1.21) | 0.496 |
| 20-100% Poverty | Ref. |  |
| Unknown | 1.08 (0.68 – 1.73) | 0.756 |
| Hospital Accreditation |  |  |
| Non-CoC-Accredited | Ref. |  |
| CoC-accredited | 0.74 (0.59 – 0.93) | 0.010 |
| Primary Site |  |  |
| Right colon | Ref. |  |
| Transverse colon | 0.76 (0.54 – 1.06) | 0.103 |
| Left colon | 0.82 (0.65 – 1.03) | 0.091 |
| Overlapping lesion of colon and colon NOS | 1.78 (1.11 – 2.86) | 0.017 |

NH = Non-Hispanic, NOS = Not otherwise specified, CoC = Commission on Cancer

**Supplemental Table 5.** Multivariable adjusted odds ratio for receipt of guideline concordant lymphadenectomy and chemotherapy measures among patients with low socioeconomic status or residing in a rural/urban location.

|  | **Lymphadenectomy for Stage I-III, OR (95% CI)** | **p-value** | **Chemotherapy for Stage III-IV, OR (95% CI)** | **p-value** |
| --- | --- | --- | --- | --- |
| Sex |  |  |  |  |
| Male | Ref. |  | Ref. |  |
| Female | 1.12 (1.06 – 1.19) | <0.001 | 1.07 (1.01 – 1.14) | 0.033 |
| Age |  |  |  |  |
| 18-49 | 1.88 (1.63 – 2.17) | <0.001 | 2.37 (2.09 – 2.69) | <0.001 |
| 50-59 | 1.18 (1.08 – 1.30) | <0.001 | 1.40 (1.28 – 1.54) | <0.001 |
| 60-69 | Ref. |  | Ref. |  |
| 70-79 | 0.93 (0.86 – 1.01) | 0.090 | 0.64 (0.59 – 0.69) | <0.001 |
| ≥80 | 0.82 (0.75 – 0.89) | <0.001 | N/A |  |
| Insurance Status |  |  |  |  |
| Medicare | Ref. |  | Ref. |  |
| Private | 1.12 (1.02 – 1.22) | 0.041 | 1.38 (1.26 – 1.52) | <0.001 |
| Medicaid | 1.00 (0.88 – 1.13) | 0.493 | 0.99 (0.88 – 1.12) | 0.713 |
| Not insured | 0.99 (0.82 – 1.18) | 0.553 | 0.78 (0.67 – 0.90) | <0.001 |
| Other government | 1.17 (0.93 – 1.46) | 0.210 | 1.07 (0.85 – 1.34) | 0.340 |
| Unknown | 0.95 (0.86 – 1.04) | 0.058 | 0.76 (0.69 – 0.84) | <0.001 |
| Race and Ethnicity |  |  |  |  |
| NH White | Ref. |  | Ref. |  |
| NH Black | 0.83 (0.77 – 0.90) | <0.001 | 0.76 (0.71 – 0.82) | 0.045 |
| NH American Indian/Alaska Native | 0.94 (0.72 – 1.23) | 0.694 | 0.99 (0.75 – 1.29) | 0.137 |
| NH Asian or Pacific Islander | 1.28 (1.01 – 1.62) | <0.001 | 0.71 (0.57 – 0.89) | 0.008 |
| Hispanic (All Races) | 0.91 (0.83 – 1.00) |  | 0.76 (0.69 – 0.84) | 0.005 |
| Hospital Accreditation |  |  |  |  |
| Non-CoC-Accredited | Ref. |  | Ref. |  |
| CoC-accredited | 1.97 (1.87 – 2.09) | <0.001 | 2.03 (1.90 – 2.16) | <0.001 |
| Stage |  |  |  |  |
| I | Ref. |  | N/A |  |
| II | 1.85 (1.72 – 2.00) | <0.001 | N/A |  |
| III | 1.84 (1.73 – 1.97) | <0.001 | Ref. |  |
| IV | N/A |  | 0.77 (0.73 – 0.82) | <0.001 |
| Primary Site |  |  |  |  |
| Right colon | Ref. |  | Ref. |  |
| Transverse colon | 0.47 (0.43 – 0.51) | <0.001 | 0.89 (0.80 – 0.99) | 0.014 |
| Left colon | 0.40 (0.37 – 0.42) | <0.001 | 1.06 (0.99 – 1.14) | <0.001 |
| Overlapping lesion of colon and colon NOS | 0.52 (0.44 – 0.61) | 0.020 | 0.44 (0.40 – 0.49) | <0.001 |

NH = Non-Hispanic, NOS = Not otherwise specified, CoC = Commission on Cancer
